# Criteria for Diagnosis of Polycystic Ovary Syndrome during Adolescence: Literature Review

**DOI:** 10.3390/diagnostics12081931

**Published:** 2022-08-10

**Authors:** Alexia S. Peña, Ethel Codner, Selma Witchel

**Affiliations:** 1 Discipline of Paediatrics, The University of Adelaide Robinson Research Institute, 72 King William Road, Adelaide, SA 5006, Australia; 2 Endocrinology and Diabetes Department, Women’s and Children’s Hospital, 72 King William Road, Adelaide, SA 5006, Australia; 3 Institute of Child and Maternal Research, School of Medicine, University of Chile, Santiago 836-0160, Chile; 4 UPMC Children’s Hospital of Pittsburgh, University of Pittsburgh, Pittsburgh, PA 15224, USA

**Keywords:** adolescents, girls, polycystic ovary syndrome, diagnosis

## Abstract

Polycystic ovary syndrome (PCOS) is one of the most common endocrine conditions in women. PCOS may be more challenging to diagnose during adolescence due to an overlap with the physiological events of puberty, which are part of the diagnostic criteria in adult women. This review focuses on the evidence available in relation to PCOS diagnostic criteria for adolescents. Adolescent PCOS should be diagnosed using two main criteria irregular -menstrual cycles (relative to number of years post-menarche) and hyperandrogenism (clinical and/or biochemical); after excluding other conditions that mimic PCOS. Accurate definitions of the two main criteria will decrease challenges/controversies with the diagnosis and provide timely diagnosis during adolescence to establish early management. Despite the attempts to create accurate diagnostic criteria and definitions, this review highlights the limited research in this area, especially in the follow up of adolescents presenting with one diagnostic feature that are called “at risk of PCOS”. Studies in adolescents continue to use the Rotterdam diagnostic criteria that uses pelvic ultrasound. This is inappropriate, because previous and emerging data that show many healthy adolescents have polycystic ovarian morphology in the early years post-menarche. In the future, anti-Müllerian hormone levels might help support PCOS diagnosis if adolescents meet two main criteria.

## 1. Introduction

Polycystic ovary syndrome (PCOS) is one of the most common endocrine conditions, affecting 8–13% [1] of women and 3.4–19.6% of adolescent girls, depending on the diagnostic criteria used and population studied [2,3,4,5,6]. The highest prevalence (19.6%) was reported in adolescents with Type 2 diabetes [6]. PCOS is also a familial condition with twin studies indicating that hereditability is approximately 70% [7]. Positive family history of PCOS in first degree relative has been reported in 24% of adolescents with PCOS and is higher in adolescents with PCOS compared to healthy adolescents [8,9]. Over 20 genetic loci associated with PCOS were identified according to genome-wide association studies among different ethnic populations of women [10,11,12,13]. Daughters of women with PCOS have been reported to have a five-fold increased risk of developing PCOS [14].

The World Health Organization defines adolescence as the period between 10 and 19 years of age, which includes critical changes in growth, puberty, and development. These physiological changes, including menstrual irregularities, hyperandrogenism, and polycystic ovarian morphology (PCOM) overlap with adult diagnostic criteria of PCOS, making diagnosis during adolescence challenging and controversial [15,16,17,18]. The first diagnostic criteria for PCOS in adult women were established by a consensus meeting at the National Institutes of Health (NIH) in 1990 [19] and was followed by multiple consensuses, statements and/or guidelines for adult women with limited acknowledgment of the difficulties for diagnosing PCOS in adolescents [20,21,22,23]. A recent systematic review identified 13 clinical practice guidelines for diagnosis and management of PCOS with seven of those covering adolescent PCOS and highlighting the variability in the scope of the guidelines and methodologies used which may influence translation to clinical practice [24]. Over the last decade, there have been three international adolescent consensuses/guidelines supporting the use of NIH PCOS diagnostic criteria. These documents include two main diagnostic criteria: menstrual irregularities/ovulatory dysfunction and hyperandrogenism once other conditions that mimic PCOS have been excluded (Table 1) [17,19,25,26]. The 2003 Rotterdam criteria for PCOS diagnosis was not recommended in the adolescent PCOS guidelines as it is based on the presence of two of three features: menstrual irregularities, clinical or biochemical hyperandrogenism, and PCOM on ultrasound; and PCOM should not be used in adolescents [20]. The adolescent consensuses/guidelines aimed to decreased the variability on diagnosis criteria used [3,27] and highlighted the lack of longitudinal data on natural history of PCOS during adolescence [17,25,26].

There is a need for a careful approach and diagnostic criteria to provide timely diagnosis during adolescence [17,28]. Appropriate early diagnosis will enable timely management of lifelong health comorbidities associated with PCOS such as type 2 diabetes, cardio-metabolic abnormalities, non-alcoholic fatty liver disease, and psychological comorbidities; and ensure that adolescents are suitably transitioned to adult care [29,30,31]. The diagnostic criteria should avoid “over diagnosis” that causes unnecessary concerns about future fertility or other complications; and at the same time highlights the need for follow up of adolescents “at risk” of PCOS who do not fulfill the diagnostic criteria [17,25,32]. Recent quality or care improvement studies have highlighted the importance of education on adolescent PCOS diagnostic criteria to improve care [33,34]. The aim of this manuscript was to review the evidence on diagnostic criteria available for adolescents with PCOS to guide timely and appropriate diagnosis of these adolescents. This review does not include the diagnosis and management of comorbidities associated with PCOS.

## 2. Search Strategy

**Figure 1:**
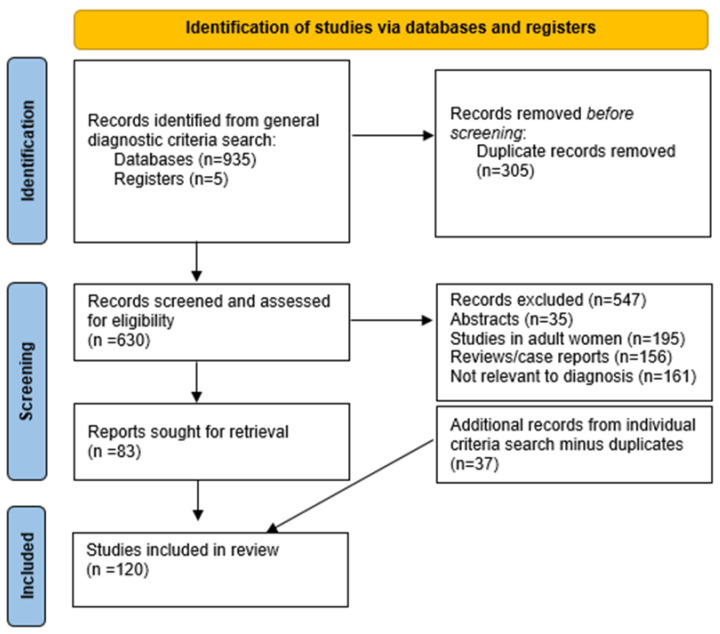
PRISMA search flow algorithm.

The following databases were searched: Ovid MEDLINE, Embase, EBM Reviews, Cochrane Central Register of Controlled Trials, EBM Reviews-Cochrane Database of Systematic Reviews, and Cumulative Index to Nursing and Allied Health Literature (CINAHL), up to March 2022. The search terms for the literature search are included in Appendix A. The search strategy followed the PRISMA model, which is shown in Figure 1. The searches performed highlighted a large number of studies in adult women, studies not relevant to PCOS diagnosis and reviews/case reports which were excluded unless they were international consensuses or evidence-based. This review included original studies in adolescents, systematic reviews and meta-analysis, population-based studies (both in selected and unselected populations), consensus papers, and international guidelines.

## 3. Main Criteria to Diagnose PCOS during Adolescence

### 3.1. Menstrual Cycle Irregularity and Ovulatory Dysfunction

Oligomenorrhea and anovulation are a cornerstone element of the diagnosis of PCOS in adult women. The first diagnostic criteria of PCOS, the NIH criteria, used oligomenorrhea/amenorrhea as a required element to diagnose PCOS in adult women [19]. A systematic review evaluating diagnostic criteria for diagnosis of PCOS during adolescence, demonstrated that almost all the studies require menstrual irregularities to be present for the diagnosis of PCOS in adolescents [16]. However, special criteria should be used to define menstrual irregularity in adolescents (Table 1) [17]. Additionally, primary amenorrhea or the lack of menstruation within three years of thelarche is a feature of adolescent PCOS within the criterion of menstrual cycle irregularities. Several studies in adult women have used the presence of anovulation as a criterion for the diagnosis of PCOS, which may be a physiologic event occurring in some menstrual cycles in the early post-menarcheal years.

In the years that follow menarche, regular menstrual cyclicity may take some time to be attained. During puberty, the gonadal axis is activated in a progressive way and the achievement of menarche does not signal a full maturation of the hormonal feedback on the hypothalamic-pituitary-ovary axis [35,36]. The frequent presence of anovulatory cycles and menstrual irregularities observed in early adolescence has been explained by the absence of the physiologic positive estrogen feedback stimulating the mid cycle luteinizing hormone (LH) surge which is required for ovulation [37]. However, immaturity in the follicle stimulating hormone (FSH) and ovarian responses have also been shown to have a role [35,36].

The American Academy of Pediatrics and American College of Obstetrics and Gynecology published criteria to define menstrual abnormalities for adolescents [38,39]. and suggested that the presence of persistent menstrual cycles longer than 45 days during the six years following menarche should be considered to be oligomenorrhea. These data were based on the fact that ninety percent of cycles are within the range of 21–45 days, and cycles longer than 90 days represent the 95th percentile for length [40,41,42]. Another element to be considered for evaluation of menstrual cyclicity in adolescents is that a higher variability in the duration of the menstrual cycles is observed in young compared to adult women [43]. However, recent studies showed that most adolescents attain regular menstrual cycles with a similar duration of adult women after two to three years post-menarche. An Italian study evaluated menstrual cycles in 3783 adolescents attending schools and showed that after 3–4 years post-menarche less than 10% of the adolescents present cycles longer than 35 days and shorter than 21 days (polymenorrhagia) [44]. Similarly, only 6% of adolescents aged 16 years showed persistent menstrual cycles longer than 35 days in a Danish cohort [45,46]. Moreover, adolescents with oligomenorrhea at the age of 15 years show a tendency to persist at the age of 18 years [47] and at the age of 26 years [48]. Based on these data, there is consensus data that there are difficulties diagnosing PCOS the years following menarche. Two international studies that reported on the diagnosis of PCOS during adolescence recommended waited two years after menarche to diagnose oligomenorrhea if persistent cycles longer than 45 days were present [25,26]. However, an international evidence-based study suggested that adolescent menstrual irregularities may be diagnosed when persistent menstrual cycles longer than 45 days, present in the 1–3 years post-menarche, and after this period the <21 days and >35 days should be used (Table 1) [17], which is similar to the criteria used in adult women [49]. In addition, if a menstrual cycle is longer than 90 days one year post-menarche, it is also a sign of menstrual irregularity (Table 1) [17].

Anovulation is another aspect that differs in adolescent girls compared to adult women. In healthy young women, only 10% of the cycles are anovulatory [50,51]. However, studies evaluating ovulation in the years following puberty have shown ovulation in only 20% of the menstrual cycles during the first year post-menarche [52], 25–35% in the second year [52,53], 45% in the fourth year [52], and reaching around 70% of the cycles between 5–9 years post-menarche [53,54]. Nevertheless, another study that evaluated ovulation in young healthy women recruited in colleges aged 16–24 years showed that one third of the cycles may be anovulatory [55]. Therefore, the determination of ovulation by serum progesterone levels in a single menstrual cycle, a method that has been used for the diagnosis of PCOS in adult women [20], is not recommended in adolescents.

Another noteworthy difference between adolescent girls and adult women is that oligomenorrhea has been used as an index of the presence of anovulation in the latter group. In adolescents, menstrual cycle irregularities do not necessarily indicate the presence of anovulation [55] and a large proportion of healthy adolescents with irregular menstrual cycles are still ovulating despite irregular and infrequent menses [56]. A similar lack of correlation between menstrual cycle duration and ovulation has been reported in adolescents with type 1 diabetes [57].

Several studies showed that the presence of oligomenorrhea in adolescents is associated with hyperandrogenism. Adolescents with oligomenorrhea (>42 days) at the age of 14 years had higher free testosterone and dehydroepiandrosterone sulfate (DHEAS) [58]. Similarly, when using the 35 days criteria for diagnosing menstrual irregularity at the age of 16 years, an evaluation of 317 Danish adolescents showed that they had higher androgen levels [45,46]. Moreover, a Finnish study that evaluated 2448 adolescents (age 16 years) showed that adolescents with oligomenorrhea had higher testosterone and free testosterone levels compared to regularly menstruating adolescents [59]. Similar data were reported in a large Dutch study that evaluated 14–16 year old adolescents [60]. Recently, it was reported that the risk of having elevated androgen levels in oligomenorrheic girls is increased in obese adolescents [61].

The presence of menstrual irregularities are associated with higher body mass index (BMI), higher blood pressure, and lower insulin sensitivity [58,62]. A prospective study showed that adolescents who have three or more menstrual cycles longer than 42 days at the age of 14 years had higher BMI, insulin, glucose levels, and insulin resistance at the age of 25 years [58], suggesting that even menstrual irregularities at a young age suggest a higher metabolic risk later as a young adult.

**Figure 2:**
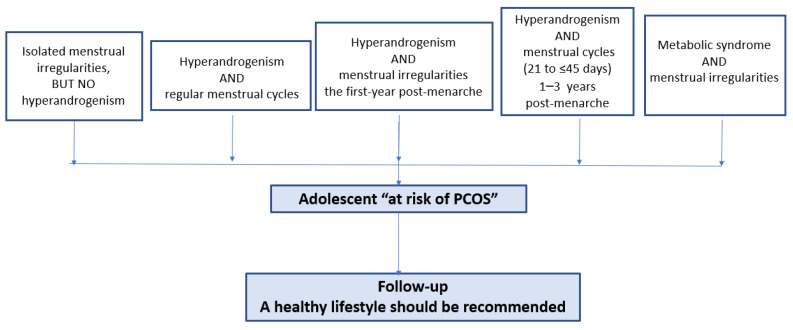
Definitions of adolescents “at risk of PCOS”.

The importance of menstrual irregularities during adolescence as a marker of future risk of PCOS was recently reported in a long follow up study of adolescents in a Dutch cohort. Caanen et al. followed a group of 271 adolescents from the age of 15 years of age of whom 30% had oligoamenorrhea and found that the risk of developing PCOS was 22.5% in the group with oligoamenorrhea compared with 5% in the group that had regular menstrual cycles [63]. Similar data were reported in studies published in an epidemiologic Finnish study [48]. Therefore, adolescents that present with isolated irregular menstrual cycles or menstrual cycles that are not considered irregular according to time postmenarche can be defined as “at risk of PCOS” and require follow up (Figure 2).

### 3.2. Hyperandrogenism

Hyperandrogenism is typically categorized as clinical or biochemical. Hirsutism and acne are considered to be manifestations of clinical hyperandrogenism that require a comprehensive physical examination. The fact that many adolescents develop mild features of clinical hyperandrogenism during puberty confounds the diagnosis of PCOS.

The semi-objective scoring system, the Ferriman Gallwey score may be used to characterize the extent of the hirsutism but should take into account if hair removal methods have been used. The score will be affected if laser/electrolysis, waxing methods, or shaving has been used in the previous 3 months, 4 weeks, and 5 days, respectively [64]. The modified Ferriman Gallwey (mFG) score involves the assessment of nine body areas (upper lip, chin, neck, chest, upper and lower abdomen, thighs, upper and lower back) with scoring between 0–4 depending on the extent of terminal hair growth (rigid hair more than 5 mm in length). However, the optimal cut point to define hirsutism likely depends on ethnic background with higher cut offs described in Mongoloid Asian compared to White and Black women [65,66]. Of note, there are no studies defining the optimal cut off for adolescents of different ethnicities and mild hirsutism may reflect ethnic variation or normal pubertal progression rather than indicating hyperandrogenism during adolescence. Nevertheless, the cutaneous findings need to be interpreted within the clinical context of a specific patient. Based on the international evidence-based guidelines, a mFG greater than 4–6 may be consistent with hirsutism [30,67]. A cross-sectional study of 154 adolescents two years post-menarche in Canada including 60 with PCOS according to Rotterdam criteria, 48 who were classified as at risk of PCOS by authors but fulfill NIH PCOS criteria, and 46 healthy controls showed mean mFG of 17.1, 15.9, and 5.7, respectively. The presence of hirsutism and acne was similar among the adolescents with PCOS diagnosed using Rotterdam or NIH criteria [68]. Lower mean mFG scores (6–8.5) were reported in cohorts of adolescents with PCOS in the USA including Hispanics and black adolescents [69,70]. Hirsutism defined as mFG score higher than 6 and higher than 8 was reported in 60–70% and 50% of adolescents with PCOS respectively [8,68,70]. Higher hirsutism scores are related to higher testosterone levels according to population and cross-sectional studies of adolescents [59,60,69,71,72]. Hirsutism must also be distinguished from hypertrichosis, which is defined as excessive vellus hair distributed in a non-sexual pattern.

Mild to moderate acne is common among adolescents and among adolescents with PCOS [73,74]. However, when acne is more severe, PCOS should be considered to be a diagnosis [17,25,26]. There is no consensus on a single score for evaluation of the severity of acne, but in general adolescents that have larger number of comedonal lesions that are resistant to topical medications and cause scaring have severe acne. A recent systematic review and meta-analysis showed that the prevalence of acne in women with PCOS is higher compared to women without PCOS (43 vs. 21%) highlighting higher prevalence in East Asia. Additionally, this study showed that the estimated prevalence of acne was higher in adolescents with PCOS compared to women with PCOS (59 vs. 42%) [74]. Both acne and hirsutism are the most common skin manifestations of adolescents with PCOS [68,70].

Another feature of clinical hyperandrogenism is the female pattern hair loss or previously called alopecia. This is a diffuse thinning of scalp hair around the crown area that can be present in 28% of women with PCOS [75]. There are no studies specifically evaluating female pattern hair loss in adolescents with PCOS. Two studies from the Middle East including 53 and 55 adolescents with PCOS, respectively, reported only one adolescent with PCOS and female pattern hair loss (1.8%) [8,9].

In relation to biochemical hyperandrogenism, all reports stress the importance of sensitive and consistent testosterone assays. Radioimmunoassays were used to measure total testosterone but more recently, liquid chromatography-mass spectroscopy methods were developed [76]. However, reference intervals defining normative data in adolescent girls are lacking and hormone concentrations vary during the peripubertal years. An additional consideration when measuring androgen levels in any woman is that should be in the absence of hormonal contraception for at least three months to avoid interference with results.

Nicolaides and colleagues recommended use of free testosterone using a reliable assay, free androgen index, and bioavailable testosterone as measure of biochemical hyperandrogenism [77]. Specific recommendations for total testosterone concentration range from 55 ng/dL (1.9 nmol/L) [25]. Khashchenko et al. reported androgen concentrations in 130 adolescents aged 15–17 years and two years post-menarche diagnosed with PCOS by Rotterdam criteria. Median hormone concentrations were testosterone 55 ng/dL (1.9 nmol/L) (range 35–72 ng/dL (1.2–2.5 nmol/L)) and androstenedione concentrations 15.8 ng/mL (55.2 nmol/L) (range 11.6–23.3 ng/mL (40.5–81.3 nmol/L)). For DHEAS, the mean± standard deviation was 6.8 ± 3.2 µmol/L [78]. These investigators determined that using cut-points for testosterone > 33 ng/dL (1.15 nmol/L), androstenedione > 11.45 ng/mL (40 nmol/L), and LH/FSH ratio > 1.23 showed sensitivity of 63.2–78.2% and specificity of 84.4–93.7% in PCOS diagnosis in their sample. It is important to recognize that assays differ resulting in different androgen values. Asanidze et al. reported that around 50% of adolescents with PCOS according to Rotterdam criteria and NIH criteria have biochemical hyperandrogenism but the cut off values used were not reported in the study [68]. Adolescents with higher free androgen index at 15–16 years of age are more likely to develop PCOS at the age 26 years [48].

Despite the fact that isolated clinical hyperandrogenism and biochemical hyperandrogenism occur in 16.1% and 6.6% of adolescents, respectively, only 1.3% have both clinical and biochemical hyperandrogenism according to a large cross-sectional population study of 16 to 19 years old girls in Italy [72]. Adolescents with isolated hyperandrogenism (clinical and/or biochemical) and regular menstrual cycles should not be diagnosed with PCOS but should be considered “at risk of PCOS” (Figure 2).

### 3.3. Other Investigations and/or Features Not Part of the Diagnostic Criteria

Other investigations and/or features that are not part of the criteria for the adolescent PCOS diagnosis are included in this section. These are helpful to rule out other conditions causing menstrual cycle irregularities and/or hyperandrogenism; and/or comorbidities associated with PCOS and include blood tests, pelvic ultrasound, anti-Müllerian hormone (AMH), and insulin resistance [17,25,26]. 

Some blood tests are essential to diagnose PCOS in adolescent and adult women for the exclusion of other disorders that can cause irregular menstrual cycles and/or hyperandrogenism including beta human chorionic gonadotropin hormone (if sexually active), LH, FSH, thyroid function tests, prolactin, midnight salivary cortisol, and 17-hydroxyprogesterone (17-OHP) [79,80]. Demirci et al. investigated whether any other indicator could distinguish PCOS from non-classic congenital adrenal hyperplasia. They concluded that measuring 17-OHP was essential to differentiating between PCOS and non-classic congenital adrenal hyperplasia [81]. No differences in heterozygosity for *CYP21A2* variants was found among adolescent diagnosed with PCOS, adolescents at risk of PCOS, or healthy controls; however, one limitation of this study is that it does not appear that V281L mutation was assayed [82]. Androstenedione can be also elevated in non-classical adrenal hyperplasia. Mildly elevated DHEAS can be observed in adolescents with PCOS [58], but very high DHEAS levels are more likely to indicate the presence of an androgen secreting tumor [83,84].

#### 3.3.1. Pelvic Ultrasound to Evaluate PCOM

**Figure 3:**
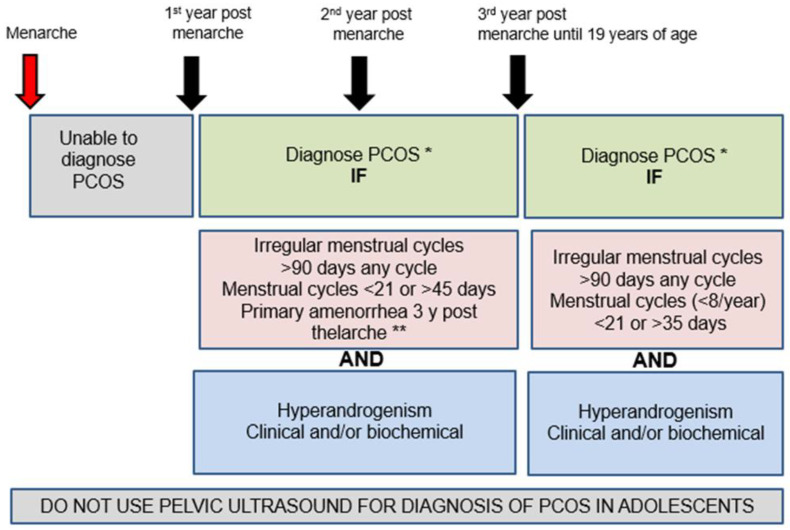
Adolescent PCOS diagnosis according to time post-menarche. * After other conditions that mimic PCOS have been excluded. ** Further investigations might be required to evaluate primary amenorrhea.

Even though pelvic ultrasound and PCOM are part of the Rotterdam diagnostic criteria for PCOS in adult women [20] it is not recommended for the diagnosis of PCOS during adolescence as it can cause over diagnosis of PCOS during this life stage [3,32]. This is supported by previous evidence summarized in adolescent international guidelines [17,25,30] and more recent evidence (Figure 3) [32,45,85]. Please note that the international evidence-based guidelines recommended not to use pelvic ultrasound for the diagnosis of PCOS in those with gynecological age of <8 years [17].

There are two main reasons for avoiding the use of pelvic ultrasound during adolescence. The first one is the fact that the majority of ultrasounds are made trans-abdominally not trans-vaginally, affecting the accuracy of findings [17]. There are two studies that used a trans-rectal ultrasound in adolescents. One study showed higher mean ovarian volume (9.2 vs. 4.4 cm^3^) in 69 adolescents diagnosed with PCOS according to NIH criteria compared to 26 healthy adolescents and reported that a mean ovarian volume of 6.74 cm^3^ had a 92.3% specificity and 75.4% sensitivity to distinguish PCOS in Chinese adolescents [86]. This study did not evaluate ovarian follicle count another component of PCOM. The second study showed that trans-rectal ultrasound was more reliable than trans-abdominal ultrasound evaluating PCOM but it also highlighted that healthy adolescents also had PCOM [87]. This study also showed that ovarian stromal to total area ratio was significantly higher in adolescents with PCOS compared to healthy adolescents with PCOM and without PCOM. Ovarian stromal to total area ratio was most significantly correlated with androgen levels in adolescents with PCOS [87,88]. Pelvic magnetic resonance imaging in particular for overweight adolescents with PCOS can accurate estimate ovarian stromal to total area ratio and antral follicle count; however, it is not be feasible to use this imaging modality routinely [89,90].

The second reason for avoiding using pelvic ultrasound during adolescence is the presence of PCOM in healthy adolescents, which can be a transient condition [52,85,91]. There is also significant overlap of PCOM in healthy adolescents and in adolescents with PCOS [32,45,52,71,85,91,92,93,94]. PCOM has been demonstrated in healthy adolescents using transabdominal ultrasound and irrespective of the PCOM criteria used [92]. The prevalence of PCOM according to Rotterdam Consensus (ovarian volume larger than 10 cm^3^ or more than 12 follicles [20]) was 34.3%; according to Androgen Excess-PCOS Society (ovarian volume larger than 10 cm^3^ [95]) was 25.3%; and according to the international adolescent PCOS consensus (ovarian volume larger than 12 cm^3^ [25]) was 12.8% [92]. Higher prevalence of PCOS up to 57% has been reported using Rotterdam criteria in healthy adolescents [85]. A recent cross-sectional population-based study of 257 healthy adolescents showed that PCOM with normal ovarian stromal to total area ratio is more likely to occur 1–3 years post-menarche and PCOM with increased stromal to total area ratio more likely to occur four years after menarche [85]. The presence of PCOM is higher in adolescents with irregular menstrual cycles compared to healthy girls from a population-based study. [45] Recent studies that included adolescents at least two years post menarche using Rotterdam criteria for PCOS diagnosis during adolescence demonstrated no difference in ovarian volume but higher antral follicle count between adolescents with PCOS and healthy controls [68]. In contrast, Khaschenko and Assens showed both higher ovarian volume and antral follicle count in adolescents with PCOS [45,78].

Pelvic ultrasound can be used to evaluate other possible uterine or ovarian abnormalities in adolescents that present with primary amenorrhoea [96].

#### 3.3.2. Anti-Müllerian Hormone (AMH)

AMH is a glycoprotein of the transforming growth factor beta family secreted by granulosa cells of developing ovarian follicles in females. AMH levels increase through childhood in healthy females before declining with age later in life [97,98,99,100]. AMH has been related to ovarian follicle count and it is considered a marker of ovarian reserve [45,101]. Elevated AMH levels relate to PCOM in non-obese adolescents with regular menstrual cycles [92,102].

The use of serum AMH as a single test for diagnosis of PCOS in women or adolescents is not currently recommended due to heterogeneity between studies in relation to age, assays used and PCOS diagnosis criteria used. Studies showed an important overlap in values in women with and without PCOS [17,30,103]. A review and a recent study using the international evidence-based guidelines for PCOS diagnosis in 154 adolescents support the use of AMH as an additional diagnostic marker for adolescents at risk of PCOS [68,104].

Some studies evaluating AMH in the diagnosis of adolescent PCOS have used Rotterdam criteria for PCOS diagnosis, which is not appropriate for adolescents [8,9,78,105,106,107,108]. The following studies have used PCOS NIH diagnosis criteria of irregular menstrual cycles and hyperandrogenism with inconsistent results in relation to AMH levels in adolescents with PCOS [109,110,111,112]. AMH levels are higher in non-obese [109] and obese adolescents with PCOS [111,113,114] and AMH levels decreased with weight loss and other treatments in adolescents with PCOS [68,114,115].

Few studies in adolescents have determined AMH cut off values for PCOS diagnosis with variable sensitivities and specificities, which can increase with the addition of other PCOS features such as total testosterone levels [78,111,116]. Cut offs reported included AMH values of 5.8 ng/mL (41.4 pmol/L) [9], 5.95 ng/mL (42.5 pmol/L) [109], 6.26 ng/mL (44.7 pmol/L) ([111], 6.32 ng/mL (45.1 pmol/L) [116] and 7.2 ng/mL (51.8 pmol/L) [78,110]. These cut offs are higher compared to the cut offs reported in a large cohort of women with PCOS [117]. An AMH of 3.15 ng/mL (22.5 pmol/L) at 16 years of age predicted PCOS at 26 years of age diagnosed by both NIH and Rotterdam criteria in a population-based cohort study [59]. This is in contrast to a recent longitudinal cohort study that reported that adolescent AMH levels were not a prognostic marker for PCOS in adult women [63].

AMH levels alone may not be able to be used as criteria for adolescent PCOS diagnosis but might help supporting the diagnosis if adolescents meet both irregular menstrual cycles and hyperandrogenism criteria.

#### 3.3.3. Insulin Resistance

Despite that insulin resistance as manifested by acanthosis nigricans and higher insulin levels occur commonly in adolescents with PCOS and it is exacerbated by obesity; this is not recommended for the diagnosis of PCOS during adolescence [25,26,29]. On the other hand, the presence of insulin resistance should reinforce the screening of adolescents for type 2 diabetes as a comorbidity [118]. There is a high incidence of type 2 diabetes in adolescents with PCOS [6,119] and both diabetes and PCOS increase risk of other comorbidities such as depression during adolescence [120].

Adolescents with insulin resistance and other features of metabolic syndrome require healthy lifestyle advice irrespective of PCOS diagnosis during adolescence (Figure 2).

## 4. Discussion and Conclusions

PCOS diagnosis during adolescence is more challenging and controversial due to an overlap with physiological events of puberty, which are part of the diagnostic criteria in adult women. The only criterion that applies to adolescents from all adult diagnostic criteria is the exclusion of other conditions that mimic PCOS. This review summarized the available evidence in relation to PCOS diagnostic criteria for adolescents highlighting the need for using two main criteria (NIH criteria): the first one is the presence of irregular menstrual cycles which must be well defined according to the number of years post-menarche and the second one is hyperandrogenism (clinical and/or biochemical) (Figure 3). Additionally, pelvic ultrasound and PCOM should not be used as criterion for adolescent PCOS diagnosis, which precludes the use of PCOS Rotterdam diagnostic criteria during adolescence. There is a potential for using AMH levels to support PCOS diagnosis if adolescents meet two main criteria. The research including adolescents who meet only one of the PCOS diagnostic criteria either irregular menstrual cycles or hyperandrogenism is limited at present but these adolescents should be considered “at risk of PCOS” and ongoing follow up should be established with reinforcement of healthy lifestyle (Figure 2). Longitudinal research tracking physiological events of puberty from menarche will clarify the trajectory of symptoms of adolescents “at risk of PCOS” and review if in some adolescents, we may be too early to make the diagnosis or we may be missing an opportunity for diagnosis around the time of transition of care to adult physicians.

## Figures and Tables

**Table 1:** Current specific consensus/guidelines criteria for diagnosis of PCOS during adolescence.

Criteria Definition	Witchel S et al. 2015 [25]	Ibanez L et al. 2017 [26]	Pena AS et al. 2020 [17]
Menstrual Irregularity Ovulatory dysfunction	Menstrual cycles < 20 days and >45 days two years post-menarche	Irregular cycles two years post-menarche	Strict definition according to time post-menarche Irregular cycles are normal 1st year post-menarche Menstrual cycles < 21 and >45 days 1–3 years post-menarche Menstrual cycles < 21 days and >35 days 3 years post-menarche (<8 cycles per year)
	Menstrual cycles > 90 days 1 year post-menarche	Menstrual cycles > 90 days 1 year post-menarche	Menstrual cycles > 90 days 1 year post-menarche
	Primary amenorrhea by 15 years or after 2–3 years post thelarche	Primary amenorrhea in girls that completed puberty	Primary amenorrhea by 15 years or after 3 years post thelarche
Hyperandrogenism	Clinical: moderate to severe hirsutism (no definition provided) and/or persistent acne unresponsive to topical therapy Rarely alopecia Biochemical: confirmation test in girls with hyperandrogenism Persistent elevation of total testosterone and/or free testosterone A single androgen test two standard deviations above the mean for the assay	Clinical: progressive hirsutism and/or moderate to severe acne unresponsive to topical therapy (severe cystic acne) Rarely alopecia Biochemical: confirmation test in girls with hyperandrogenism using high quality assays. No clear cut off for testosterone given	Clinical: hirsutism defined as modified Ferriman Gellway score. 4–6 and/or severe acne Rarely alopecia Biochemical: In females with irregular cycles yet without hyperandrogenism testosterone, free testosterone of free androgen index can assist with diagnosis. No cut offs given

## Data Availability

Not applicable.

## Appendix A. Search Strategies for Diagnosis as Run 4 March 2022

Database: Ovid MEDLINE(R) and Epub Ahead of Print, In-Process, In-Data-Review & Other Non-Indexed Citations and Daily <1946 to 2 March 2022>

1. Polycystic ovary syndrome/  16279
2. polycystic ovar*.mp.  21744
3. poly-cystic ovar*.mp.  51
4. (PCOS or PCOD).mp.  13672
5. (stein-leventhal or leventhal).mp.  911
6. (ovar* adj5 (sclerocystic or polycystic or poly-cystic)).mp.  21864
7. or/1-6  22558
8. Young Adult/  981588
9. Adolescent/  2161302
10. 1Child/  1819791
11. (adolescen* or teen* or school-age* or schoolage* or youth* or juvenile* or p?ediatric* or girl* or (young adj3 (adult* or person* or people* or wom#n))).ti,ab,kw.  1120473
12. or/8-11  3870344
13. *Diagnosis/  13392
14. Missed Diagnosis/  258
15. Delayed Diagnosis/  7701
16. Diagnostic Errors/  39204
17. diagnos*.ti,kf.  738538
18. (diagnos* adj6 controver*).ab.  3416
19. (diagnos* adj6 dilemma*).ab.  5049
20. (diagnos* adj6 experience*).ab.  14386
21. (diagnos* adj6 dissatisf*).ab.  150
22. (diagnos* adj6 satisf*).ab.  3611
23. (diagnos* adj6 challeng*).ab.  40293
24. (diagnos* adj2 miss*).ab.  4865
25. (diagnos* adj2 delay*).ab.  24809
26. misdiagnos*.ti,ab,kf.  38867
27. or/13-26  863236
28. 7 and 12 and 27    377
29. limit 28 to english language  326

Database: Embase <1974 to 2 March 2022>

1. ovary polycystic disease/  31017
2. polycystic ovar*.mp.  27051
3. poly-cystic ovar*.mp.  185
4. (PCOS or PCOD).mp.  20618
5. (stein-leventhal or leventhal).mp.  605
6. (ovar* adj5 (sclerocystic or polycystic or poly-cystic)).mp.  34194
7. or/1-6  36308
8. young adult/  448275
9. adolescent/  1651934
10. child/  1907656
11. (adolescen* or teen* or school-age* or schoolage* or youth* or juvenile* or p?ediatric* or girl* or (young adj3 (adult* or person* or people* or wom#n))).ti,ab,kw.  1512100
12. or/8-11  3624844
13. *diagnosis/  62017
14. missed diagnosis/  928
15. delayed diagnosis/  15408
16. diagnostic error/  64671
17. diagnos*.ti,kf.  861099
18. (diagnos* adj6 controver*).ab.  4754
19. (diagnos* adj6 dilemma*).ab.  7606
20. (diagnos* adj6 experience*).ab.  22581
21. (diagnos* adj6 dissatisf*).ab.  227
22. (diagnos* adj6 satisf*).ab.  5542
23. (diagnos* adj6 challeng*).ab.  62196
24. (diagnos* adj2 miss*).ab.  7740
25. (diagnos* adj2 delay*).ab.  39406
26. misdiagnos*.ti,ab,kf.  55961
27. or/13-26  1055751
28. 7 and 12 and 27    513
29. limit 28 to english language  454

Database: EBM Reviews-Cochrane Central Register of Controlled Trials <January 2022>

1. Polycystic ovary syndrome/  1649
2. polycystic ovar*.mp.  4330
3. poly-cystic ovar*.mp.  125
4. (PCOS or PCOD).mp.  3508
5. (stein-leventhal or leventhal).mp.  57
6. (ovar* adj5 (sclerocystic or polycystic or poly-cystic)).mp.  4491
7. or/1-6  4858
8. Young Adult/  73866
9. Adolescent/  110345
10. Child/  52129
11. (adolescen* or teen* or school-age* or schoolage* or youth* or juvenile* or p?ediatric* or girl* or (young adj3 (adult* or person* or people* or wom#n))).ti,ab,kw.  125810
12. or/8-11  262647
13. Diagnosis/  69
14. Missed Diagnosis/  9
15. Delayed Diagnosis/  28
16. Diagnostic Errors/  286
17. diagnos*.ti,kw.  64022
18. (diagnos* adj6 controver*).ab.  189
19. (diagnos* adj6 dilemma*).ab.  59
20. (diagnos* adj6 experience*).ab.  1344
21. (diagnos* adj6 dissatisf*).ab.  14
22. (diagnos* adj6 satisf*).ab.  775
23. (diagnos* adj6 challeng*).ab.  1224
24. (diagnos* adj2 miss*).ab.  300
25. (diagnos* adj2 delay*).ab.  746
26. misdiagnos*.ti,ab,kw.  599
27. or/13-26  67044
28. 7 and 12 and 27    51
29. limit 28 to english language  48

Database: EBM Reviews-Cochrane Database of Systematic Reviews <2005 to 2 March 2022>

1. polycystic ovar*.mp.  149
2. poly-cystic ovar*.mp.  0
3. (PCOS or PCOD).mp.  84
4. (stein-leventhal or leventhal).mp.  39
5. (ovar* adj5 (sclerocystic or polycystic or poly-cystic)).mp.  150
6. or/1-5  174
7. (adolescen* or teen* or school-age* or schoolage* or youth* or juvenile* or paediatric* or girl* or (young adj3 (adult* or person* or people* or wom#n))).ti,ab,kw.  1241
8. diagnos*.ti,kw.  629
9. (diagnos* adj6 controver*).ab.  1
10. (diagnos* adj6 dilemma*).ab.  0
11. (diagnos* adj6 experience*).ab.  15
12. (diagnos* adj6 dissatisf*).ab.  0
13. (diagnos* adj6 satisf*).ab.  1
14. (diagnos* adj6 challeng*).ab.  11
15. (diagnos* adj2 miss*).ab.  1
16. (diagnos* adj2 delay*).ab.  10
17. misdiagnos*.ti,ab,kw.  6
18. or/8-17    648
19. 6 and 7 and 18  0

Database: CINAHL Plus with Full Text

1. (MH “Polycystic Ovary Syndrome”)  4,429
2. polycystic ovar*    5,698
3. poly-cystic ovar*  26
4. PCOS or PCOD    4,958
5. “stein-leventhal” or Leventhal  1,267
6. ovar* N6 (sclerocystic or polycystic or poly-cystic)   5,705
7. S1 OR S2 OR S3 OR S4 OR S5 OR S6  7,733
8. (MH “Adolescence+”)  575,454
9. (MH “Child”)    503,695
10. adolescen* or teen* or child* or school-age* or schoolage* or youth* or juvenile* or pediatric* or paediatric* or girl*  1,294,856
11. S8 OR S9 OR S10  1,294,856
12. (MM “Diagnosis”)  4,700
13. (MH “Failure to Diagnose”)  1,465
14. (MH “Diagnosis, Delayed”)  4,904
15. (MH “Diagnostic Errors”)  12,392
16. TI diagnos*  137,514
17. AB diagnos* N7 controver* OR AB diagnos* N7 dilemma* OR AB diagnos* N7 experience* OR AB diagnos* N7 dissatisf* OR AB diagnos* N7 satisf* OR AB diagnos* N7 challeng* OR AB diagnos* N7 miss* OR AB diagnos* N7 delay* OR TI misdiagnos* OR AB misdiagnos* 42,186
18. S12 OR S13 OR S14 OR S15 OR S16 OR S17  184,345
19. S7 AND S11 AND S18  108
20. S7 AND S11 AND S18  Limiters-English Language  107

**PCOS diagnosis and menstrual cycle search strategy as run 16 May 2022**

**Ovid MEDLINE(R) and Epub Ahead of Print, In-Process, In-Data-Review & Other Non-Indexed Citations and Daily <1946 to 13 May 2022>**

1. Polycystic ovary syndrome/  16686
2. polycystic ovar*.mp.  22098
3. poly-cystic ovar*.mp.  52
4. (PCOS or PCOD).mp.  13942
5. (stein-leventhal or leventhal).mp.  912
6. (ovar* adj5 (sclerocystic or polycystic or poly-cystic)).mp.  22219
7. or/1-6  22911
8. Young Adult/  990846
9. Adolescent/  2175898
10. Child/  1841609
11. (adolescen* or teen* or school-age* or schoolage* or youth* or juvenile* or p?ediatric* or girl* or (young adj3 (adult* or person* or people* or wom#n))).ti,ab,kf.  1140005
12. or/8-11  3904962
13. Diagnosis/  17514
14. Diagnosis, Differential/  465318
15. Missed Diagnosis/  280
16. Delayed Diagnosis/  7853
17. Diagnostic Errors/  39330
18. diagnos*.ti,kf.  746831
19. clinical diagnosis.ab.  48824
20. differential diagnosis.ab.  103778
21. (diagnos* adj3 criteria).ab.  63792
22. (diagnos* adj6 controver*).ab.  3445
23. (diagnos* adj6 dilemma*).ab.  5122
24. (diagnos* adj6 experience*).ab.  14665
25. (diagnos* adj6 dissatisf*).ab.  151
26. (diagnos* adj6 satisf*).ab.  3659
27. (diagnos* adj6 challeng*).ab.  41398
28. (diagnos* adj2 miss*).ab.  4989
29. (diagnos* adj2 delay*).ab.  25305
30. undiagnos*.ti,ab,kf.  22874
31. or/13-30  1348101
32. Menstrual Cycle/  13873
33. Menstruation Disturbances/  7386
34. Amenorrhea/  10053
35. Oligomenorrhea/  741
36. (menstru* or menses or period*).ti,ab,kf.  1913338
37. (amenorrhea or amenorrhoea).ti,ab,kf.  14826
38. (oligomenorrhea or oligomenorrhoea).ti,ab,kf.  1399
39. or/32-38  1932211
40. 7 and 12 and 31 and 39  311
41. limit 40 to (english or spanish)  274

**Embase <1974 to 13 May 2022>**

1. ovary polycystic disease/  31705
2. polycystic ovar*.mp.  27648
3. poly-cystic ovar*.mp.  189
4. (PCOS or PCOD).mp.  21164
5. (stein-leventhal or leventhal).mp.  607
6. (ovar* adj5 (sclerocystic or polycystic or poly-cystic)).mp.  34931
7. or/1-6  37109
8. young adult/  457981
9. adolescent/  1668346
10. child/  1930245
11. (adolescen* or teen* or school-age* or schoolage* or youth* or juvenile* or p?ediatric* or girl* or (young adj3 (adult* or person* or people* or wom#n))).ti,ab,kw.  1531909
12. or/8-11  3666415
13. diagnosis/  1370526
14. differential diagnosis/  356015
15. missed diagnosis/  991
16. delayed diagnosis/  15697
17. diagnostic error/  65252
18. diagnos*.ti,kf.  871654
19. clinical diagnosis.ab.  72479
20. differential diagnosis.ab.  150575
21. (diagnos* adj3 criteria).ab.  103665
22. (diagnos* adj6 controver*).ab.  4794
23. (diagnos* adj6 dilemma*).ab.  7711
24. (diagnos* adj6 experience*).ab.  22955
25. (diagnos* adj6 dissatisf*).ab.  229
26. (diagnos* adj6 satisf*).ab.  5604
27. (diagnos* adj6 challeng*).ab.  63546
28. (diagnos* adj2 miss*).ab.  7918
29. (diagnos* adj2 delay*).ab.  40023
30. undiagnos*.ti,ab,kf.  35267
31. or/13-30  2596598
32. menstrual cycle/  38625
33. menstruation/  20937
34. exp menstruation disorder/  69557
35. (menstru* or menses or period*).ti,ab,kf.  2625182
36. (amenorrhea or amenorrhoea).ti,ab,kf.  18642
37. (oligomenorrhea or oligomenorrhoea).ti,ab,kf.  2181
38. or/32-37  2687373
39. 7 and 12 and 31 and 38  656
40. limit 39 to (english or spanish)  606

**EBM Reviews-Cochrane Central Register of Controlled Trials <April 2022>**

1. Polycystic ovary syndrome/  1679
2. polycystic ovar*.mp.  4297
3. poly-cystic ovar*.mp.  132
4. (PCOS or PCOD).mp.  3496
5. (stein-leventhal or leventhal).mp.  57
6. (ovar* adj5 (sclerocystic or polycystic or poly-cystic)).mp.  4464
7. or/1-6  4827
8. Young Adult/  74694
9. Adolescent/  111127
10. Child/  52868
11. (adolescen* or teen* or school-age* or schoolage* or youth* or juvenile* or p?ediatric* or girl* or (young adj3 (adult* or person* or people* or wom#n))).ti,ab,kw.  125587
12. or/8-11  263322
13. Diagnosis/  68
14. Diagnosis, Differential/  1490
15. Missed Diagnosis/  11
16. Delayed Diagnosis/  28
17. Diagnostic Errors/  288
18. diagnos*.ti,kw.  65057
19. clinical diagnosis.ab.  5116
20. differential diagnosis.ab.  788
21. (diagnos* adj3 criteria).ab.  16717
22. (diagnos* adj6 controver*).ab.  105
23. (diagnos* adj6 dilemma*).ab.  45
24. (diagnos* adj6 experience*).ab.  760
25. (diagnos* adj6 dissatisf*).ab.  6
26. (diagnos* adj6 satisf*).ab.  474
27. (diagnos* adj6 challeng*).ab.  806
28. (diagnos* adj2 miss*).ab.  204
29. (diagnos* adj2 delay*).ab.  560
30. undiagnos*.ti,ab,kw.  1143
31. or/13-30  87378
32. Menstrual Cycle/  869
33. Menstruation Disturbances/  235
34. Amenorrhea/  343
35. Oligomenorrhea/  46
36. (menstru* or menses or period*).ti,ab,kw.  281778
37. (amenorrhea or amenorrhoea).ti,ab,kw.  2311
38. (oligomenorrhea or oligomenorrhoea).ti,ab,kw.  361
39. or/32-38  283399
40. 7 and 12 and 31 and 39  30
41. limit 40 to (english or spanish)  29

**EBM Reviews-Cochrane Database of Systematic Reviews <2005 to 11 May 2022>**

1. polycystic ovar*.mp.  149
2. poly-cystic ovar*.mp.  0
3. (PCOS or PCOD).mp.  84
4. (stein-leventhal or leventhal).mp.  39
5. (ovar* adj5 (sclerocystic or polycystic or poly-cystic)).mp.  150
6. or/1-5  174
7. (adolescen* or teen* or school-age* or schoolage* or youth* or juvenile* or p?ediatric* or girl* or (young adj3 (adult* or person* or people* or wom#n))).ti,ab,kw.  1253
8. diagnos*.ti,ab,kw.  1727
9. undiagnos*.ti,ab,kw.  16
10. or/8-9  1731
11. (menstru* or menses or period*).ti,ab,kw.  1345
12. (amenorrhea or amenorrhoea).ti,ab,kw.  44
13. (oligomenorrhea or oligomenorrhoea).ti,ab,kw.  2
14. or/11-13  1353
15. 6 and 7 and 10 and 14  3

**PCOS diagnosis and Hyperandrogenism search strategy as run 16 May 2022**

**Ovid MEDLINE(R) and Epub Ahead of Print, In-Process, In-Data-Review & Other Non-Indexed Citations and Daily <1946 to 13 May 2022>**

1. Polycystic ovary syndrome/  16686
2. polycystic ovar*.mp.  22098
3. poly-cystic ovar*.mp.  52
4. (PCOS or PCOD).mp.  13942
5. (stein-leventhal or leventhal).mp.  912
6. (ovar* adj5 (sclerocystic or polycystic or poly-cystic)).mp.  22219
7. or/1-6  22911
8. Young Adult/  990846
9. Adolescent/  2175898
10. Child/  1841609
11. (adolescen* or teen* or school-age* or schoolage* or youth* or juvenile* or p?ediatric* or girl* or (young adj3 (adult* or person* or people* or wom#n))).ti,ab,kf.  1140005
12. or/8-11  3904962
13. Diagnosis/  17514
14. Diagnostic Techniques, Endocrine/  1071
15. Biomarkers/  329596
16. Diagnosis, Differential/  465318
17. Missed Diagnosis/  280
18. Delayed Diagnosis/  7853
19. Diagnostic Errors/  39330
20. diagnos*.ti,kf.  746831
21. (biomarker* or marker*).ti,ab,kf.  1148168
22. clinical diagnosis.ab.  48824
23. differential diagnosis.ab.  103778
24. diagnostic definition*.ab.  312
25. (diagnos* adj3 criteria).ab.  63792
26. (diagnos* adj6 controver*).ab.  3445
27. (diagnos* adj6 dilemma*).ab.  5122
28. (diagnos* adj6 experience*).ab.  14665
29. (diagnos* adj6 dissatisf*).ab.  151
30. (diagnos* adj6 satisf*).ab.  3659
31. (diagnos* adj6 challeng*).ab.  41398
32. (diagnos* adj2 miss*).ab.  4989
33. (diagnos* adj2 delay*).ab.  25305
34. undiagnos*.ti,ab,kf.  22874
35. or/13-34  2541274
36. Hyperandrogenism/  2277
37. exp Dehydroepiandrosterone/  11984
38. hyperandrogen*.ti,ab,kf.  6042
39. ((androgen or testosterone) adj2 (increas* or elevate* or raise* or high*)).ti,ab,kf.  10782
40. Hirsutism/  4159
41. hirsutism.ti,kf.  1908
42. Acne Vulgaris/  12765
43. (acne adj2 (severe or vulgaris)).ti,kf.  3481
44. Alopecia/  12264
45. alopecia.ti,kf.  10073
46. or/36-45  59877
47. 7 and 12 and 35 and 46  638
48. limit 47 to (english or spanish)  597

**Embase <1974 to 13 May 2022>**

1. ovary polycystic disease/  31705
2. polycystic ovar*.mp.  27648
3. poly-cystic ovar*.mp.  189
4. (PCOS or PCOD).mp.  21164
5. (stein-leventhal or leventhal).mp.  607
6. (ovar* adj5 (sclerocystic or polycystic or poly-cystic)).mp.  34931
7. or/1-6  37109
8. young adult/  457981
9. adolescent/  1668346
10. child/  1930245
11. (adolescen* or teen* or school-age* or schoolage* or youth* or juvenile* or p?ediatric* or girl* or (young adj3 (adult* or person* or people* or wom#n))).ti,ab,kw.  1531909
12. or/8-11  3666415
13. diagnosis/  1370526
14. endocrine system examination/  545
15. biological marker/  381458
16. differential diagnosis/  356015
17. missed diagnosis/  991
18. delayed diagnosis/  15697
19. diagnostic error/  65252
20. diagnos*.ti,kf.  871654
21. (biomarker* or marker*).ti,ab,kf.  1654043
22. clinical diagnosis.ab.  72479
23. differential diagnosis.ab.  150575
24. (diagnos* adj3 criteria).ab.  103665
25. (diagnos* adj6 controver*).ab.  4794
26. (diagnos* adj6 dilemma*).ab.  7711
27. (diagnos* adj6 experience*).ab.  22955
28. (diagnos* adj6 dissatisf*).ab.  229
29. (diagnos* adj6 satisf*).ab.  5604
30. (diagnos* adj6 challeng*).ab.  63546
31. (diagnos* adj2 miss*).ab.  7918
32. (diagnos* adj2 delay*).ab.  40023
33. undiagnos*.ti,ab,kf.  35267
34. or/13-33  4124534
35. hyperandrogenism/  8276
36. prasterone/  15819
37. hyperandrogen*.ti,ab,kf.  9102
38. ((androgen or testosterone) adj2 (increas* or elevate* or raise* or high*)).ti,ab,kf.  13861
39. hirsutism/  12014
40. hirsutism.ti,kf.  2388
41. acne vulgaris/  11465
42. (acne adj2 (severe or vulgaris)).ti,kf.  4583
43. Alopecia/  45479
44. alopecia.ti,kf.  12487
45. or/35-44  109088
46. 7 and 12 and 34 and 45  900
47. limit 46 to (english or spanish)  845

**EBM Reviews-Cochrane Central Register of Controlled Trials <April 2022>**

1. Polycystic ovary syndrome/  1679
2. polycystic ovar*.mp.  4297
3. poly-cystic ovar*.mp.  132
4. (PCOS or PCOD).mp.  3496
5. (stein-leventhal or leventhal).mp.  57
6. (ovar* adj5 (sclerocystic or polycystic or poly-cystic)).mp.  4464
7. or/1-6  4827
8. Young Adult/  74694
9. Adolescent/  111127
10. Child/  52868
11. (adolescen* or teen* or school-age* or schoolage* or youth* or juvenile* or p?ediatric* or girl* or (young adj3 (adult* or person* or people* or wom#n))).ti,ab,kw.  125587
12. or/8-11  263322
13. Diagnosis/  68
14. Diagnostic Techniques, Endocrine/  47
15. Biomarkers/  15881
16. Diagnosis, Differential/  1490
17. Missed Diagnosis/  11
18. Delayed Diagnosis/  28
19. Diagnostic Errors/  288
20. diagnos*.ti,kw.  65057
21. (biomarker* or marker*).ti,ab,kw.  84953
22. clinical diagnosis.ab.  5116
23. differential diagnosis.ab.  788
24. diagnostic definition*.ab.  9
25. (diagnos* adj3 criteria).ab.  16717
26. (diagnos* adj6 controver*).ab.  105
27. (diagnos* adj6 dilemma*).ab.  45
28. (diagnos* adj6 experience*).ab.  760
29. (diagnos* adj6 dissatisf*).ab.  6
30. (diagnos* adj6 satisf*).ab.  474
31. (diagnos* adj6 challeng*).ab.  806
32. (diagnos* adj2 miss*).ab.  204
33. (diagnos* adj2 delay*).ab.  560
34. undiagnos*.ti,ab,kw.  1143
35. or/13-34  172054
36. Hyperandrogenism/  146
37. exp Dehydroepiandrosterone/  694
38. hyperandrogen*.ti,ab,kw.  859
39. ((androgen or testosterone) adj2 (increas* or elevate* or raise* or high*)).ti,ab,kw.  1044
40. Hirsutism/  204
41. hirsutism.ti,kw.  449
42. Acne Vulgaris/  1473
43. (acne adj2 (severe or vulgaris)).ti,kw.  1985
44. Alopecia/  630
45. alopecia.ti,kw.  2918
46. or/36-45  8608
47. 7 and 12 and 35 and 46  65
48. limit 47 to (english or spanish)  62

**EBM Reviews-Cochrane Database of Systematic Reviews <2005 to 11 May 2022>**

1. polycystic ovar*.mp.  149
2. poly-cystic ovar*.mp.  0
3. (PCOS or PCOD).mp.  84
4. (stein-leventhal or leventhal).mp.  39
5. (ovar* adj5 (sclerocystic or polycystic or poly-cystic)).mp.  150
6. or/1-5  174
7. (adolescen* or teen* or school-age* or schoolage* or youth* or juvenile* or p?ediatric* or girl* or (young adj3 (adult* or person* or people* or wom#n))).ti,ab,kw.  1253
8. diagnos*.ti,ab,kw.  1727
9. undiagnos*.ti,ab,kw.  16
10. or/8-9  1731
11. hyperandrogen*.ti,ab,kw.  6
12. ((androgen or testosterone) adj2 (increas* or elevate* or raise* or high*)).ti,ab,kw.  1
13. hirsutism.ti,kw.  8
14. (acne adj2 (severe or vulgaris)).ti,kw.  14
15. alopecia.ti,kw.  6
16. or/11-14  22
17. 6 and 7 and 10 and 16  1

**PCOS diagnosis and pelvic ultrasound PCOM search strategy as run 24 March 2022**

**Ovid MEDLINE(R) and Epub Ahead of Print, In-Process, In-Data-Review & Other Non-Indexed Citations and Daily <1946 to 22 March 2022>**

1. Polycystic ovary syndrome/  16385
2. polycystic ovar*.mp.  21847
3. poly-cystic ovar*.mp.  52
4. (PCOS or PCOD).mp.  13742
5. (stein-leventhal or leventhal).mp.  911
6. (ovar* adj5 (sclerocystic or polycystic or poly-cystic)).mp.  21969
7. or/1-6  22659
8. Young Adult/  985418
9. Adolescent/  2166028
10. Child/  1825339
11. (adolescen* or teen* or school-age* or schoolage* or youth* or juvenile* or p?ediatric* or girl* or (young adj3 (adult* or person* or people* or wom#n))).ti,ab,kf.  1128017
12. or/8-11  3881789
13. Diagnosis/  17509
14. Diagnostic Techniques, Endocrine/  1070
15. Diagnosis, Differential/  464734
16. Missed Diagnosis/  267
17. Delayed Diagnosis/  7754
18. Diagnostic Errors/  39243
19. diagnos*.ti,kf.  740720
20. clinical diagnosis.ab.  48220
21. differential diagnosis.ab.  103059
22. diagnostic technique*.ab.  10773
23. (diagnos* adj3 criteria).ab.  63184
24. (diagnos* adj6 controver*).ab.  3425
25. (diagnos* adj6 dilemma*).ab.  5065
26. (diagnos* adj6 experience*).ab.  14465
27. (diagnos* adj6 dissatisf*).ab.  150
28. (diagnos* adj6 satisf*).ab.  3625
29. (diagnos* adj6 challeng*).ab.  40566
30. (diagnos* adj2 miss*).ab.  4898
31. (diagnos* adj2 delay*).ab.  24943
32. undiagnos*.ti,ab,kf.  22641
33. or/13-32  1346318
34. Ultrasonography/  193782
35. Ultrasonography, Doppler/  16855
36. Imaging, Three-Dimensional/  78894
37. ultraso*.ti,ab,kf.  423835
38. doppler.ti,ab,kf.  110117
39. echograph*.ti,ab,kf.  10075
40. sonogra*.ti,ab,kf.  59806
41. imaging.ti,ab,kf.  952478
42. polycystic ovarian morphology.ti,ab,kf.  256
43. PCOM.ti,ab,kf.  316
44. or/34-43  1470075
45. 7 and 12 and 33 and 44  197
46. limit 45 to (english or spanish)  180

**Embase <1974 to 22 March 2022>**

1. ovary polycystic disease/  31135
2. polycystic ovar*.mp.  27137
3. poly-cystic ovar*.mp.  185
4. (PCOS or PCOD).mp.  20677
5. (stein-leventhal or leventhal).mp.  605
6. (ovar* adj5 (sclerocystic or polycystic or poly-cystic)).mp.  34324
7. or/1-6  36441
8. young adult/  451020
9. adolescent/  1656206
10. child/  1913414
11. (adolescen* or teen* or school-age* or schoolage* or youth* or juvenile* or p?ediatric* or girl* or (young adj3 (adult* or person* or people* or wom#n))).ti,ab,kw.  1516782
12. or/8-11  3635590
13. diagnosis/  1367804
14. differential diagnosis/  354368
15. missed diagnosis/  941
16. delayed diagnosis/  15509
17. diagnostic error/  64843
18. diagnos*.ti,kf.  863628
19. clinical diagnosis.ab.  71731
20. differential diagnosis.ab.  149450
21. diagnostic technique*.ab.  13979
22. (diagnos* adj3 criteria).ab.  102817
23. (diagnos* adj6 controver*).ab.  4766
24. (diagnos* adj6 dilemma*).ab.  7622
25. (diagnos* adj6 experience*).ab.  22680
26. (diagnos* adj6 dissatisf*).ab.  227
27. (diagnos* adj6 satisf*).ab.  5564
28. (diagnos* adj6 challeng*).ab.  62534
29. (diagnos* adj2 miss*).ab.  7784
30. (diagnos* adj2 delay*).ab.  39586
31. undiagnos*.ti,ab,kf.  34880
32. or/13-31  2589857
33. echography/  351726
34. ultrasound/  203032
35. doppler ultrasonography/  8482
36. three-dimensional imaging/  104412
37. ultraso*.ti,ab,kf.  624906
38. doppler.ti,ab,kf.  165439
39. echograph*.ti,ab,kf.  13252
40. sonogra*.ti,ab,kf.  84906
41. imaging.ti,ab,kf.  1338544
42. polycystic ovarian morphology.ti,ab,kf.  356
43. PCOM.ti,ab,kf.  502
44. or/33-43  2148774
45. 7 and 12 and 32 and 44  422
46. limit 45 to (english or spanish)  395

**EBM Reviews-Cochrane Central Register of Controlled Trials <January 2022>**

1. Polycystic ovary syndrome/  1649
2. polycystic ovar*.mp.  4330
3. poly-cystic ovar*.mp.  125
4. (PCOS or PCOD).mp.  3508
5. (stein-leventhal or leventhal).mp.  57
6. (ovar* adj5 (sclerocystic or polycystic or poly-cystic)).mp.  4491
7. or/1-6  4858
8. Young Adult/  73866
9. Adolescent/  110345
10. Child/  52129
11. (adolescen* or teen* or school-age* or schoolage* or youth* or juvenile* or p?ediatric* or girl* or (young adj3 (adult* or person* or people* or wom#n))).ti,ab,kw.  125810
12. or/8-11  262647
13. Diagnosis/  69
14. Diagnostic Techniques, Endocrine/  47
15. Diagnosis, Differential/  1491
16. Missed Diagnosis/  9
17. Delayed Diagnosis/  28
18. Diagnostic Errors/  286
19. diagnos*.ti,kw.  64022
20. diagnostic technique*.ab.  225
21. (diagnos* adj6 controver*).ab.  189
22. (diagnos* adj6 dilemma*).ab.  59
23. (diagnos* adj6 experience*).ab.  1344
24. (diagnos* adj6 dissatisf*).ab.  14
25. (diagnos* adj6 satisf*).ab.  775
26. (diagnos* adj6 challeng*).ab.  1224
27. (diagnos* adj2 miss*).ab.  300
28. (diagnos* adj2 delay*).ab.  746
29. undiagnos*.ti,ab,kw.  1141
30. or/13-29  68839
31. Ultrasonography/  5104
32. Ultrasonography, Doppler/  610
33. Imaging, Three-Dimensional/  1198
34. ultraso*.ti,ab,kw.  45636
35. doppler.ti,ab,kw.  11283
36. echograph*.ti,ab,kw.  6197
37. sonogra*.ti,ab,kw.  4359
38. imaging.ti,ab,kw.  53978
39. polycystic ovarian morphology.ti,ab,kw.  27
40. PCOM.ti,ab,kw.  20
41. or/31-40  105019
42. 7 and 12 and 30 and 41  14
43. limit 42 to english language  13

**EBM Reviews-Cochrane Database of Systematic Reviews <2005 to 16 March 2022>**

1. polycystic ovar*.mp.  149
2. poly-cystic ovar*.mp.  0
3. (PCOS or PCOD).mp.  84
4. (stein-leventhal or leventhal).mp.  39
5. (ovar* adj5 (sclerocystic or polycystic or poly-cystic)).mp.  150
6. or/1-5  174
7. (adolescen* or teen* or school-age* or schoolage* or youth* or juvenile* or p?ediatric* or girl* or (young adj3 (adult* or person* or people* or wom#n))).ti,ab,kw.  1243
8. diagnos*.ti,ab,kw.  1715
9. ultraso*.ti,ab,kw.  240
10. doppler.ti,ab,kw.  37
11. echograph*.ti,ab,kw.  0
12. sonogra*.ti,ab,kw.  9
13. imaging.ti,ab,kw.  230
14. polycystic ovarian morphology.ti,ab,kw.  0
15. PCOM.ti,ab,kw.  0
16. or/9-15    413
17. 6 and 7 and 8 and 16  0

**PCOS diagnosis and AMH search strategy as run 16 May 2022**

**Ovid MEDLINE(R) and Epub Ahead of Print, In-Process, In-Data-Review & Other Non-Indexed Citations and Daily <1946 to 13 May 2022>**

1. Polycystic ovary syndrome/  16686
2. polycystic ovar*.mp.  22098
3. poly-cystic ovar*.mp.  52
4. (PCOS or PCOD).mp.  13942
5. (stein-leventhal or leventhal).mp.  912
6. (ovar* adj5 (sclerocystic or polycystic or poly-cystic)).mp.  22219
7. or/1-6  22911
8. Young Adult/  990846
9. Adolescent/  2175898
10. Child/  1841609
11. (adolescen* or teen* or school-age* or schoolage* or youth* or juvenile* or p?ediatric* or girl* or (young adj3 (adult* or person* or people* or wom#n))).ti,ab,kf.  1140005
12. or/8-11  3904962
13. Diagnosis/  17514
14. Diagnostic Techniques, Endocrine/  1071
15. Biomarkers/  329596
16. Diagnosis, Differential/  465318
17. Missed Diagnosis/  280
18. Delayed Diagnosis/  7853
19. Diagnostic Errors/  39330
20. diagnos*.ti,kf.  746831
21. (biomarker* or marker*).ti,ab,kf.  1148168
22. clinical diagnosis.ab.  48824
23. differential diagnosis.ab.  103778
24. (diagnos* adj3 criteria).ab.  63792
25. (diagnos* adj6 controver*).ab.  3445
26. (diagnos* adj6 dilemma*).ab.  5122
27. (diagnos* adj6 experience*).ab.  14665
28. (diagnos* adj6 dissatisf*).ab.  151
29. (diagnos* adj6 satisf*).ab.  3659
30. (diagnos* adj6 challeng*).ab.  41398
31. (diagnos* adj2 miss*).ab.  4989
32. (diagnos* adj2 delay*).ab.  25305
33. undiagnos*.ti,ab,kf.  22874
34. or/13-33  2541115
35. Anti-Mullerian Hormone/  3755
36. Anti-Mullerian Hormone.ti,ab,kf.  4463
37. antimullerian hormone.ti,ab,kf.  582
38. AMH.ti,ab,kf.  5049
39. Mullerian inhibiting substance.ti,ab,kf.  579
40. or/35-39  7018
41. 7 and 12 and 34 and 40  171
42. limit 41 to (english or spanish)  167

**Embase <1974 to 13 May 2022>**

1. ovary polycystic disease/  31705
2. polycystic ovar*.mp.  27648
3. poly-cystic ovar*.mp.  189
4. (PCOS or PCOD).mp.  21164
5. (stein-leventhal or leventhal).mp.  607
6. (ovar* adj5 (sclerocystic or polycystic or poly-cystic)).mp.  34931
7. or/1-6  37109
8. young adult/  457981
9. adolescent/  1668346
10. child/  1930245
11. (adolescen* or teen* or school-age* or schoolage* or youth* or juvenile* or p?ediatric* or girl* or (young adj3 (adult* or person* or people* or wom#n))).ti,ab,kw.  1531909
12. or/8-11  3666415
13. diagnosis/  1370526
14. endocrine system examination/  545
15. biological marker/  381458
16. differential diagnosis/  356015
17. missed diagnosis/  991
18. delayed diagnosis/  15697
19. diagnostic error/  65252
20. diagnos*.ti,kf.  871654
21. (biomarker* or marker*).ti,ab,kf.  1654043
22. clinical diagnosis.ab.  72479
23. differential diagnosis.ab.  150575
24. (diagnos* adj3 criteria).ab.  103665
25. (diagnos* adj6 controver*).ab.  4794
26. (diagnos* adj6 dilemma*).ab.  7711
27. (diagnos* adj6 experience*).ab.  22955
28. (diagnos* adj6 dissatisf*).ab.  229
29. (diagnos* adj6 satisf*).ab.  5604
30. (diagnos* adj6 challeng*).ab.  63546
31. (diagnos* adj2 miss*).ab.  7918
32. (diagnos* adj2 delay*).ab.  40023
33. undiagnos*.ti,ab,kf.  35267
34. or/13-33  4124534
35. Muellerian inhibiting factor/  8673
36. Anti-Mullerian Hormone.ti,ab,kf.  6977
37. antimullerian hormone.ti,ab,kf.  996
38. AMH.ti,ab,kf.  9618
39. Mullerian inhibiting substance.ti,ab,kf.  716
40. or/35-39  13778
41. 7 and 12 and 34 and 40  192
42. limit 41 to (english or spanish)  186

**EBM Reviews-Cochrane Central Register of Controlled Trials <April 2022>**

1. Polycystic ovary syndrome/  1679
2. polycystic ovar*.mp.  4297
3. poly-cystic ovar*.mp.  132
4. (PCOS or PCOD).mp.  3496
5. (stein-leventhal or leventhal).mp.  57
6. (ovar* adj5 (sclerocystic or polycystic or poly-cystic)).mp.  4464
7. or/1-6  4827
8. Young Adult/  74694
9. Adolescent/  111127
10. Child/  52868
11. (adolescen* or teen* or school-age* or schoolage* or youth* or juvenile* or p?ediatric* or girl* or (young adj3 (adult* or person* or people* or wom#n))).ti,ab,kw.  125587
12. or/8-11  263322
13. Diagnosis/  68
14. Diagnostic Techniques, Endocrine/  47
15. Biomarkers/  15881
16. Diagnosis, Differential/  1490
17. Missed Diagnosis/  11
18. Delayed Diagnosis/  28
19. Diagnostic Errors/  288
20. diagnos*.ti,kw.  65057
21. (biomarker* or marker*).ti,ab,kw.  84953
22. clinical diagnosis.ab.  5116
23. differential diagnosis.ab.  788
24. (diagnos* adj3 criteria).ab.  16717
25. (diagnos* adj6 controver*).ab.  105
26. (diagnos* adj6 dilemma*).ab.  45
27. (diagnos* adj6 experience*).ab.  760
28. (diagnos* adj6 dissatisf*).ab.  6
29. (diagnos* adj6 satisf*).ab.  474
30. (diagnos* adj6 challeng*).ab.  806
31. (diagnos* adj2 miss*).ab.  204
32. (diagnos* adj2 delay*).ab.  560
33. undiagnos*.ti,ab,kw.  1143
34. or/13-33  172052
35. Anti-Mullerian Hormone/  134
36. Anti-Mullerian Hormone.ti,ab,kw.  580
37. antimullerian hormone.ti,ab,kw.  106
38. AMH.ti,ab,kw.  1005
39. Mullerian inhibiting substance.ti,ab,kw.  7
40. or/35-39  1214
41. 7 and 12 and 34 and 40  18
42. limit 41 to (english or spanish)  18

**EBM Reviews-Cochrane Database of Systematic Reviews <2005 to 11 May 2022>**

1. polycystic ovar*.mp.  149
2. poly-cystic ovar*.mp.  0
3. (PCOS or PCOD).mp.  84
4. (stein-leventhal or leventhal).mp.  39
5. (ovar* adj5 (sclerocystic or polycystic or poly-cystic)).mp.  150
6. or/1-5  174
7. (adolescen* or teen* or school-age* or schoolage* or youth* or juvenile* or p?ediatric* or girl* or (young adj3 (adult* or person* or people* or wom#n))).ti,ab,kw.  1253
8. diagnos*.ti,ab,kw.  1727
9. undiagnos*.ti,ab,kw.  16
10. or/8-9  1731
11. Anti-Mullerian Hormone.ti,ab,kw.  2
12. antimullerian hormone.ti,ab,kw.  0
13. AMH.ti,ab,kw.  2
14. Mullerian inhibiting substance.ti,ab,kw.  0
15. or/11-14  2
16. 6 and 7 and 10 and 15  0
